# Chemogenomic profiling in yeast reveals antifungal mode-of-action of polyene macrolactam auroramycin

**DOI:** 10.1371/journal.pone.0218189

**Published:** 2019-06-10

**Authors:** Jin Huei Wong, Mohammad Alfatah, Kiat Whye Kong, Shawn Hoon, Wan Lin Yeo, Kuan Chieh Ching, Corinna Jie Hui Goh, Mingzi M. Zhang, Yee Hwee Lim, Fong Tian Wong, Prakash Arumugam

**Affiliations:** 1 Bioinformatics Institute, Singapore; 2 Molecular Engineering Laboratory, Singapore; 3 Metabolic Engineering Research Laboratory, Institute of Chemical and Engineering Sciences, Singapore; 4 Organic Chemistry, Institute of Chemical and Engineering Sciences, Singapore; Tokyo Daigaku, JAPAN

## Abstract

In this study, we report antifungal activity of auroramycin against *Candida albicans*, *Candida tropicalis*, and *Cryptococcus neoformans*. Auroramycin, a potent antimicrobial doubly glycosylated 24-membered polyene macrolactam, was previously isolated and characterized, following CRISPR-Cas9 mediated activation of a silent polyketide synthase biosynthetic gene cluster in *Streptomyces rosesporous* NRRL 15998. Chemogenomic profiling of auroramycin in yeast has linked its antifungal bioactivity to vacuolar transport and membrane organization. This was verified by disruption of vacuolar structure and membrane integrity of yeast cells with auroramycin treatment. Addition of salt but not sorbitol to the medium rescued the growth of auroramycin-treated yeast cells suggesting that auroramycin causes ionic stress. Furthermore, auroramycin caused hyperpolarization of the yeast plasma membrane and displayed a synergistic interaction with cationic hygromycin. Our data strongly suggest that auroramycin inhibits yeast cells by causing leakage of cations from the cytoplasm. Thus, auroramycin’s mode-of-action is distinct from known antifungal polyenes, reinforcing the importance of natural products in the discovery of new anti-infectives.

## Introduction

Natural products (NPs) are a prolific source of bioactive leads with approximately 80% of clinical anti-infectives, including antifungal agents, derived from natural products [1]. Although fungal infections are on the rise, especially in expanding immuno-compromised populations [2] caused by AIDS and intensive chemotherapy cancer treatments, the number of available polyene and azole antifungal agents have largely remained the same from 1950s-1970s. A polyene macrolactone, amphotericin B is one of the leading drugs to combat serious infections due to its high potency, broad range and low frequency of resistant pathogens. Several known antifungal agents such as nystatin, filipin, and pimaricin also belong to the polyene macrolactone family. Due to growing resistance to azoles, amphotericin B is often the last line of defence for life-threatening fungal infections but its use is limited by its cytotoxicity. It is therefore a matter of paramount importance to discover antifungal agents, preferably with new modes-of-actions.

**Fig 1:**
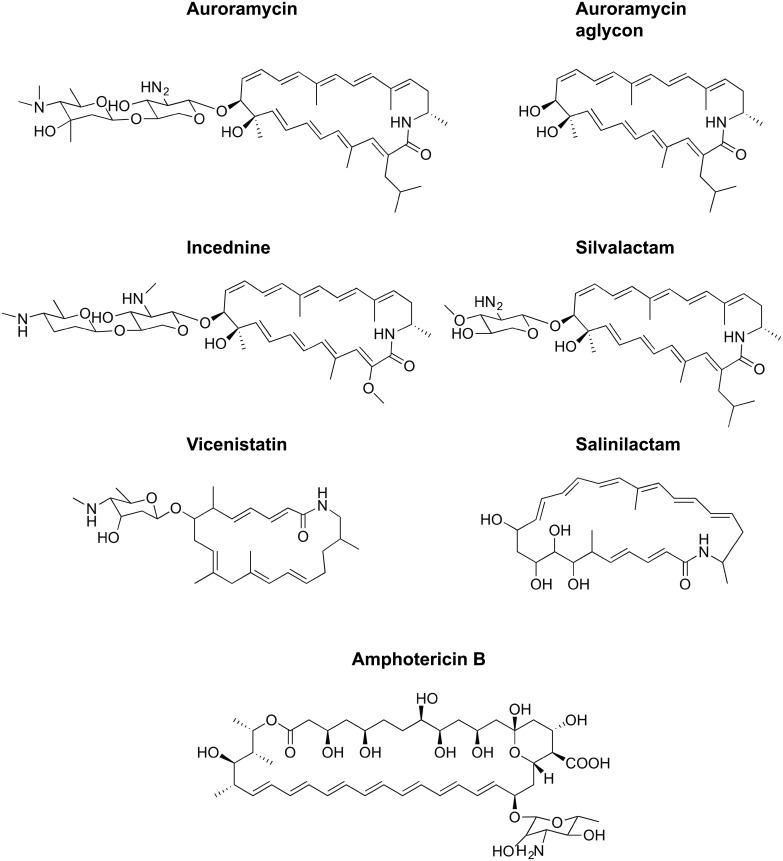
Polyene antibiotics. Chemical structures of auroramycin, auroramycin aglycon, vicenistatin, incednine, silvalactam, salinilactam, and amphotericin B.

Using a CRISPR-Cas9 mediated biosynthetic gene cluster activation strategy, we recently discovered a 24-membered doubly glycosylated polyene macrolactam, auroramycin, from *Streptomyces rosesporus* with potent activity against Gram-positive pathogens [3]. In addition to a rare isobutyrylmalonyl extender unit, auroramycin contains a unique disaccharide that is essential for its anti-bacterial bioactivity. Although the bioactivities of polyene macrolactams like vicenistatin [4], salinilactam [5], incednine [6], and silvalactam [7] (Fig 1) range from antimicrobial to anti-apoptotic modulation, none of them have been reported to have antifungal activity. In this work, we investigate the antifungal activity of auroramycin and show that its mode-of-action is distinct from that of amphotericin B.

## Results

### Auroramycin has antifungal activity

To determine their antifungal bioactivities, auroramycin and its aglycon were tested against *Candida albicans*, *Candida tropicalis*, and *Cryptococcus neoformans* (Table 1). 1–3 μM of auroramycin was sufficient to completely inhibit growth of the three fungal strains. Although auroramycin was less potent than amphotericin B in inhibiting the growth of *Candida albicans* and *Cryptococcus neoformans*, it performed slightly better against *Candida tropicalis* (Table 1). Like amphotericin B, auroramycin is cytotoxic against *S*. *cerevisiae* (S1 Fig). Highlighting the importance of the disaccharide moiety of auroramycin, the structurally similar but mono-glycosylated silvalactam (Fig 1) is inactive against yeast [7]. The auroramycin aglycon had no detectable bioactivity against all tested strains demonstrating further that the unique disaccharide motif is essential for the antifungal activity of auroramycin, (Table 1). As the first polyene macrolactam with antifungal properties, we saw the opportunity to use its activity against *Saccharomyces cerevisiae* to determine its antifungal mode-of-action.

**Table 1:** Antifungal activity of auroramycin.

Species	Strain ID	MIC, μM		
Fungi		Auroramycin	Auroramycin Aglycon	Amphotericin B
*Candida albicans*	ATCC 200918	2.52	>260	0.27
*Candida tropicalis*	ATCC 200956	1.26	>260	2.16
*Cryptococcus neoformans*	ATCC 24067	1.26–2.52	>260	0.07

Minimum Inhibitory Concentrations (MIC) of auroramycin, auroramycin-aglycon, and amphotericin B for the three fungal strains are indicated. MIC is defined as the lowest concentration of an agent that completely inhibits visible growth *in vitro* after 24 h at 30 °C for yeast.

### Homozygous profiling uncovers auroramycin-sensitive mutants

**Fig 2:**
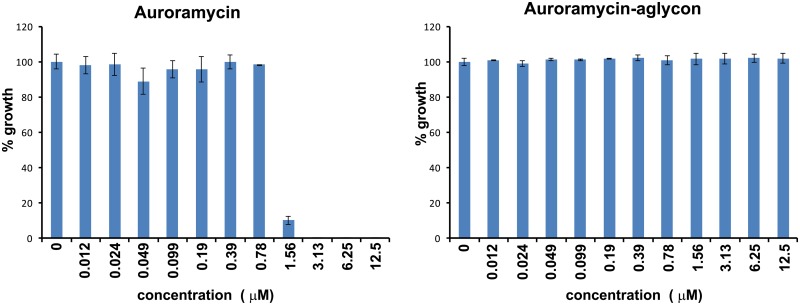
Auroramycin but not its aglycon derivative inhibits the growth of *Saccharomyces cerevisiae*. Yeast cells were exposed to auroramycin and its aglycon at the indicated concentrations in duplicates in a 96-well microplate. Cell growth was quantified by recording the absorbance at 600 nm after 24 hours. Growth (normalized with respect to DMSO-treated cells) is plotted against concentration of the compounds. Error bars (n = 2 Mean ± S.D) are indicated in the plots.

**Fig 3:**
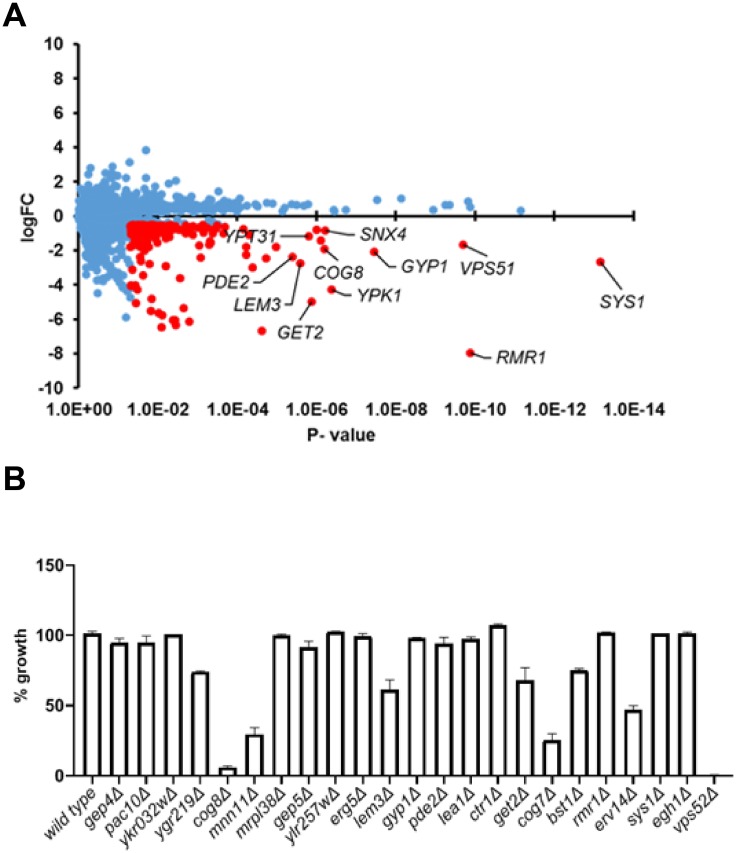
Homozygous profiling of auroramycin in *Saccharomyces cerevisiae*. (A)log FC (Fitness Coefficient) was plotted against P-value for the 4817 mutants that were analysed by HOP. Mutants with higher sensitivity to auroramycin have more negative log FC values. Individual mutants are indicated as blue circles. Mutants sensitive to auroramycin (log FC < -0.5 and P-value < 0.05) are indicated as red circles. For the sake of clarity, gene names of only a few of the top hits are shown in the plot. (B)Wild type BY4743 and 23 deletion strains that were sensitive to auroramycin in chemogenomic profiling assay, were grown in YPD-HEPES in the presence of either DMSO or auroramycin at 1.56 μM in duplicate. Normalized growth after 16 hours of incubation at 30 °C is plotted for each strain. Error bars (n = 2 Mean ± S.D) are indicated in the plots. Representative data from two independent experiments are shown in the figure.

Chemogenomic profiling in *S*. *cerevisiae* has been successfully used to determine mode-of-action of a number of antifungal compounds by identifying genes that confer resistance or sensitivity to the compound of interest [8–11]. Auroramycin completely inhibited the growth of yeast cells with an IC_50_ of 1.415 μM whereas the aglycon had no effect (Fig 2 and S2 Fig). To determine the mode-of-action of auroramycin, we performed Homozygous Profiling (HOP) in *S*. *cerevisiae* using a barcoded homozygous gene deletion library [8, 12]. The pooled yeast gene deletion library was incubated in the presence and absence of auroramycin and allowed to grow for about five generations. Next-Generation sequencing of barcodes flanking the gene deletions was performed to identify genes that confer resistance or sensitivity to auroramycin. The fitness coefficients (FC) of all 4817 deletion mutants were calculated (Fig 3A). Mutants sensitive and resistant to auroramycin are expected to have negative and positive logFC values respectively. However, mutants with positive logFC values in HOP assays exhibit little or no resistance to the compound from our previous work [13–15]. Hence, we focused on the ‘sensitive mutants’ (those with negative logFC value). To validate the HOP data, we chose 23 ‘sensitive’ mutants (*gep4Δ*, *pac10Δ*, *ykr032wΔ*, *ygr219wΔ*, *cog8Δ*, *mnn11Δ*, *mrpl38Δ*, *gep5Δ*, *ylr257wΔ*, *erg5Δ*, *lem3Δ*, *gyp1Δ*, *pde2Δ*, *lea1Δ*, *ctr1Δ*, *get2Δ*, *cog7Δ*, *bst1Δ*, *rmr1Δ*, *erv14Δ*, *sys1Δ*, *egh1Δ*, and *vps52Δ*) which were in the top 41 among 213 strains that had at least a 1.44-fold effect of growth inhibition (log FC < -0.5 and P-value < 0.05; S1 Table) and tested their growth responses to auroramycin. 39.1% of the tested mutants (9/23; *ygr219wΔ*, *cog8Δ*, *mnn11Δ*, *lem3Δ*, *get2Δ*, *cog7Δ*, *bst1Δ*, *erv14Δ*, and *vps52Δ*) showed heightened sensitivity (≥25% inhibition compared to 0% inhibition for wild type strain) to auroramycin compared to wild type after 24 hours of incubation in growth medium (Fig 3B).

### Enrichment analyses links auroramycin activity to membrane trafficking and organization

**Fig 4:**
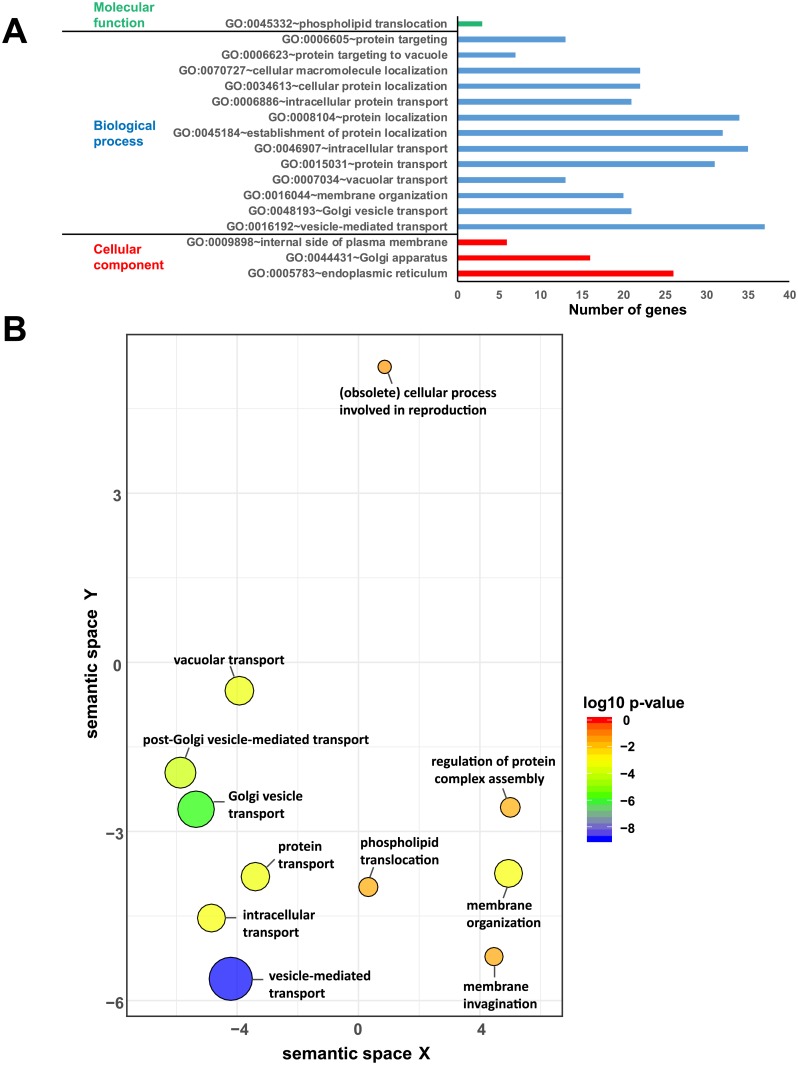
Gene Ontology (GO) analysis of genes conferring resistance to auroramycin. (A)Enrichment of GO categories in Biological process, Cellular component and Molecular function among the top cluster of genes conferring resistance to auroramycin (log FC < -0.5 and P-value < 0.05) was determined using DAVID. Number of genes in the GO category is indicated by the horizontal bar. Details of genes in each category are provided in the S1 Table. (B)Enrichment of GO categories in Biological process among the top genes conferring resistance (log FC value < -0.5 and P-value < 0.05) to auroramycin was visualized using REVIGO. Bubble colour refer to the p-value for the false discovery rates whilst circle size gives the frequency of the GO term in the underlying GO database (bubbles of more general terms are larger; http://revigo.irb.hr/).

To gain insights into auroramycin antifungal bioactivity, we performed Gene Ontology analyses of the 213 resistance genes that increase cellular sensitivity to auroramycin when deleted (log FC < -0.5 and P-value < 0.05) using the Database for Annotation, Visualization and Integrated Discovery (DAVID) [16, 17]. We used REVIGO to remove the redundant terms and visualize the data [18]. Biological process enrichment analysis revealed that a number of auroramycin resistance gene products were involved in overlapping functions such as vesicle-mediated transport (GO:0016192, 37 genes), vacuolar transport (GO:0007034, 13 genes), Golgi vesicle transport (GO:0048193, 21 genes), and membrane organization (GO:0016044, 20 genes) (Fig 4A). Cellular Component enrichment analysis indicated that several resistance gene products localized to the endoplasmic reticulum (GO:0005783, 26 genes) and a few to the internal side of plasma membrane (GO:0009898, 6 genes). Molecular function enrichment analysis identified three gene products involved in phospholipid translocation (GO:0045332, 3 genes). Visualization of GO data by REVIGO (Fig 4B) indicates that mutants defective in vacuolar/vesicular transport, Golgi vesicle transport, and membrane organization display increased sensitivity to auroramycin. Taken together, these enrichment analyses suggest that auroramycin antifungal bioactivity is associated with vesicular trafficking and membrane function.

### Auroramycin and amphotericin B inhibit yeast growth by related but distinct mechanisms

**Fig 5:**
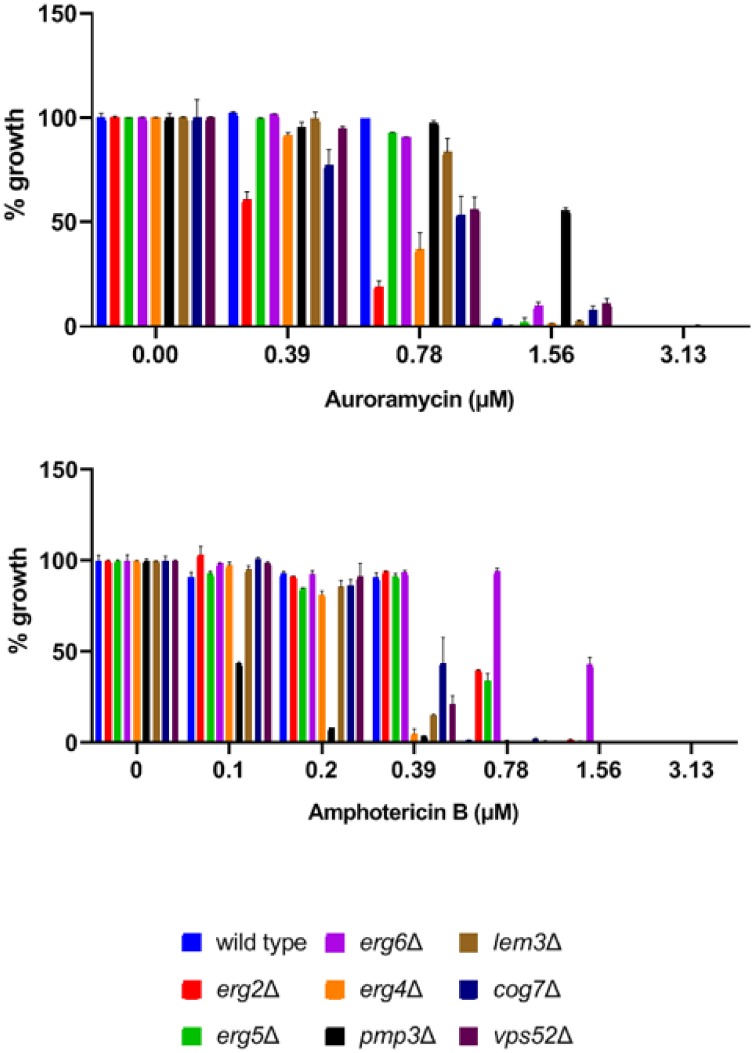
Comparison of sensitivity of mutant strains to auroramycin and amphotericin B. Growth of wild type strain BY4743 and 8 deletion strains in auroramycin or amphotericin B at the indicated concentrations in duplicate. Normalized growth after 24 hours of incubation at 30 °C is plotted for each strain. Error bars (n = 2 Mean ± S.D) are indicated in the plots.

Amphotericin B is a highly potent antifungal glycosylated polyene macrolide that inhibits yeast growth by interfering with membrane function [19]. We therefore tested whether auroramycin-sensitive mutants are also sensitive to amphotericin B. In our auroramycin-sensitivity assay, the top five sensitive mutants were *lem3Δ*, *cog7Δ*, *cog8Δ*, *vps52Δ*, and *mnn11Δ* (Fig 3B). *LEM3* encodes a subunit of the lipid flippase that translocates phospholipids to the cytosolic leaflet of the plasma membrane. *COG7*, *COG8*, *VPS52*, and *MNN11* encode Golgi-localized proteins that participate in vesicular trafficking (*COG7*, *COG8*, and *VPS52*) and N-glycosylation of proteins (*MNN11*). We chose two Golgi-related gene deletion strains, namely *cog7Δ* and *vps52Δ*, along with *lem3Δ* for our assay. We also included three amphotericin B-resistant mutants (*erg2Δ*, *erg5Δ*, and *erg6Δ)* and two amphotericin B-sensitive mutants *(erg4Δ* and *pmp3Δ*) as controls for the assay [20, 21]. We compared the sensitivities of wild type and the aforementioned eight mutant strains to auroramycin and amphotericin B. The IC_50_ values for auroramycin and amphotericin B against the wild type strain were 1.148 μM and 0.521 μM respectively (S3 Fig). As expected, the *erg2Δ*, *erg5Δ*, and *erg6Δ* mutants were resistant to amphotericin B and the *erg4Δ* and *pmp3*Δ mutants were sensitive to amphotericin B (Fig 5; bottom panel). All the three auroramycin-sensitive mutants (*lem3Δ*, *cog7Δ*, and *vps52Δ)* were also sensitive to amphotericin B (Fig 5). Surprisingly, the amphotericin B-resistant *erg2*Δ and *erg6*Δ mutants were sensitive to auroramycin and the amphotericin B-sensitive *pmp3Δ* mutant was resistant to auroramycin (Fig 5; top panel). These data suggest that auroramycin and amphotericin B exert their antifungal activities by related but distinct mechanisms.

### Ergosterol addition does not rescue auroramycin-induced toxicity

**Fig 6:**
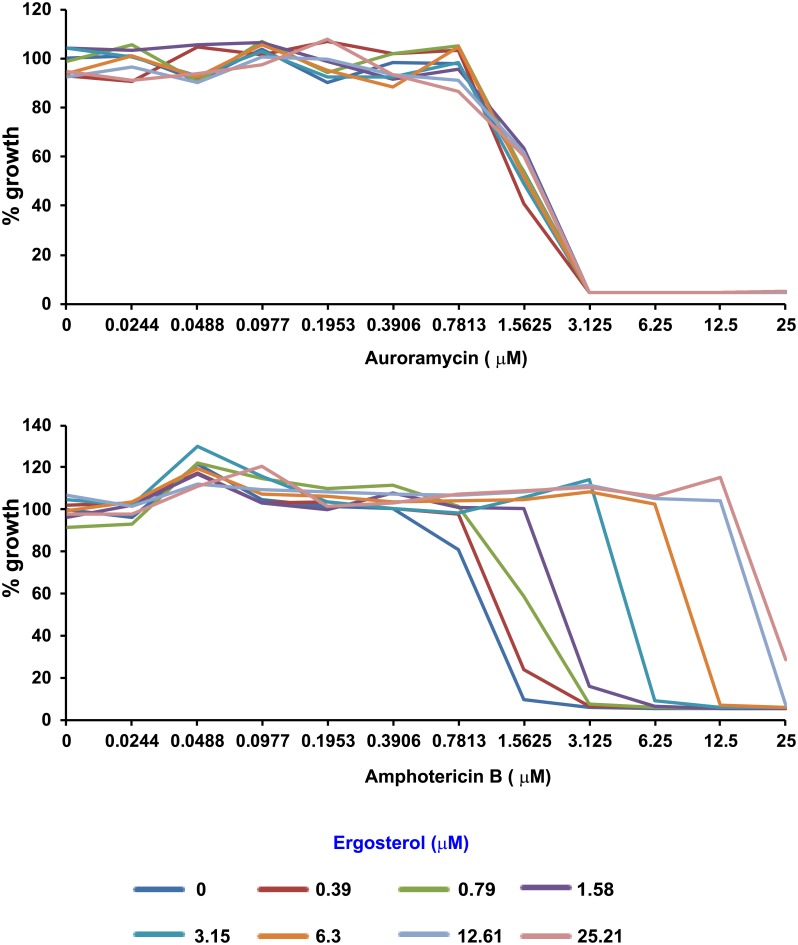
Auroramycin’s inhibitory effect is not *via* interaction with ergosterol. BY4743 cells were incubated in YPD-HEPES medium the presence of indicated concentrations of auroramycin (top) or amphotericin B (bottom) premixed with different concentrations of ergosterol in duplicate in a 96-well plate. Average growth of the duplicate cultures is plotted against the concentration of either auroramycin (upper panel) or amphotericin B (lower panel). Plots indicating the duplicate OD values with a picture of the corresponding plate are presented in S4 Fig. Representative data of one of two independent experiments are shown here.

To confirm that auroramycin and amphotericin B act via distinct mechanisms, we first determined if they bind to the same cellular target. Amphotericin B disrupts fungal membranes and causes cell death by binding to ergosterol in the membranes, which is hypothesized to be mediated by its mycoamine and carboxyl moieties and its hydrophobic polyene core [22]. Using an ergosterol competition assay [23], we explored whether auroramycin’s mode-of-action involves binding to ergosterol. Auroramycin had no detectable changes in toxicity when added to growing yeast cells in the presence of excess ergosterol (Fig 6; top panel). On the other hand, as expected of an ergosterol binding drug that will be sequestered outside the cell, toxicity of amphotericin B was reduced by exogenous ergosterol in a dose-dependent manner (Fig 6; bottom panel). These observations suggest that auroramycin does not bind to ergosterol like amphotericin B and that it has a distinct cellular target from amphotericin B.

### Auroramycin disrupts the vacuolar structure of yeast cells

**Fig 7:**
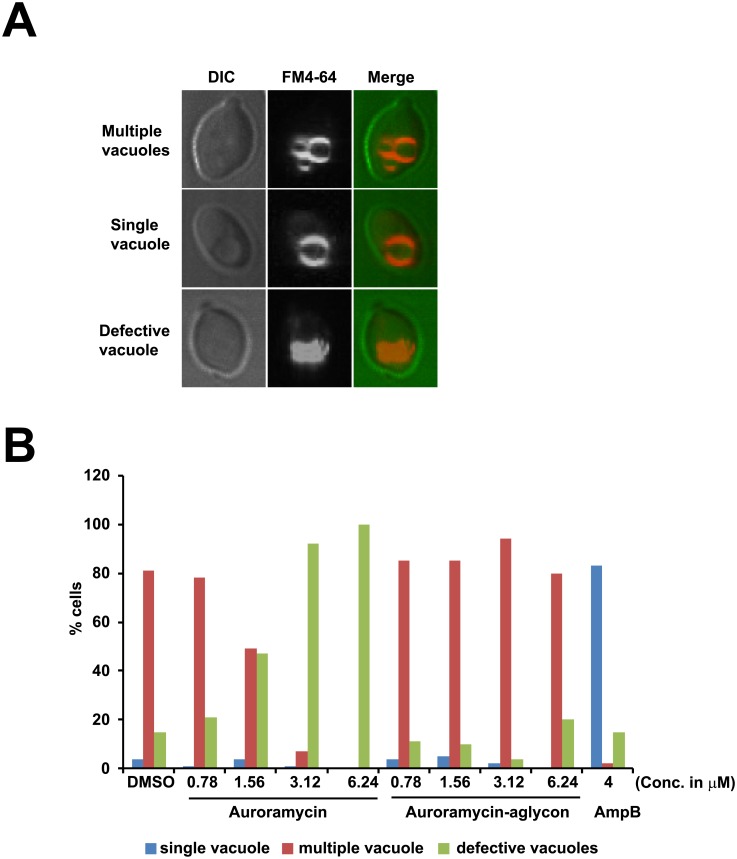
Auroramycin disrupts the vacuolar structure of yeast cells. Wild type yeast cells containing vacuoles pre-labelled with the lipophilic dye FM 4–64 were exposed to DMSO, auroramycin, auroramycin-aglycon or amphotericin B at the indicated concentrations for 1 hour. Vacuolar morphology was analysed by fluorescence microscopy. (A)Based on the FM 4–64 staining patterns, yeast cells were classified into three categories: Single vacuole or multiple vacuoles or defective vacuoles. Representative images for each category are depicted. (B)Quantification of the various categories for cells treated with auroramycin, auroramycin-aglycon or amphotericin B (n = 100 cells).

To uncover auroramycin mode-of-action, we examined the effect of auroramycin on vacuolar structure as suggested by our enrichment analyses of auroramycin resistance genes (Fig 4A). Using FM 4–64, a lipophilic dye that specifically stains the vacuolar membranes in yeast [24], we classified cells into three categories based on their vacuolar morphology: (1) a single vacuole, (2) multiple vacuoles or (3) defective vacuoles (Fig 7A) after treatment with DMSO, auroramycin or amphotericin B. Compared to the DMSO control, we observed a dose-dependence increase in cells with defective vacuoles with increasing concentrations of auroramycin (Fig 7B). At 2-fold IC_50_ and higher, 92–100% of auroramycin-treated cells contained defective vacuoles. Notably, the inactive aglycon did not have any effect on vacuolar structure. At a lethal dose of amphotericin B (> 2-fold IC_50_), 85% of cells contained a single vacuole as previously reported [25]. These experiments confirm that amphotericin B and auroramycin have different effects on vacuolar structure and suggest that auroramycin disrupts vacuolar integrity.

### Auroramycin disrupts the membrane integrity of yeast cells

**Fig 8:**
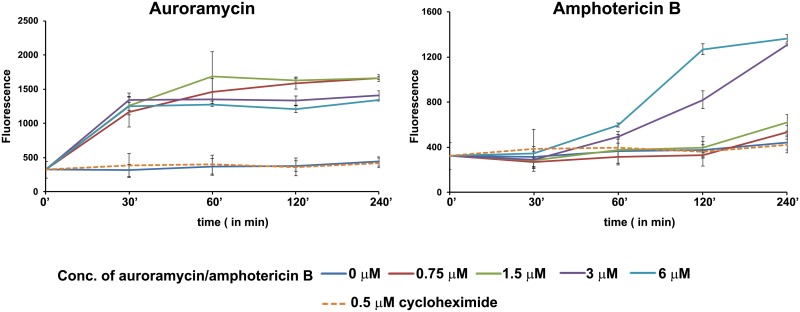
Auroramycin disrupts the membrane permeability of yeast cells. Fluorescence reading (emission at 525 nm, excitation at 495 nm) in the supernatants of CFDA-labelled wild type yeast cells at different time points, after treatment with DMSO, cycloheximide or various concentrations of auroramycin or amphotericin B in triplicates. Error bars (n = 3 Mean ± S.D) represent standard deviation. Representative data from two independent experiments are shown.

We also examined the effect of auroramycin on membrane integrity of yeast cells using carboxyfluorescein diacetate succinimidyl ester (CFDA-SE). CFDA-SE is a cell-permeable ester that is retained intracellularly following esterase cleavage [26]. In presence of auroramycin or amphotericin B but not cytostatic inhibitor cycloheximide, leakage of CFDA was observed (Fig 8). For auroramycin, CFDA leakage was observed at all concentrations, even below its IC_50_ (Fig 8: left panel). In contrast, we only observed slight CFDA leakage at concentrations for amphotericin B close to thrice its IC_50_ (Fig 8: right panel), which is consistent with the hypothesis that a threshold amphotericin B concentration is required before membrane pores can be formed [27]. These results show that auroramycin disrupts the membrane integrity of yeast cells.

### Salt suppresses the inhibitory effect of auroramycin

**Fig 9:**
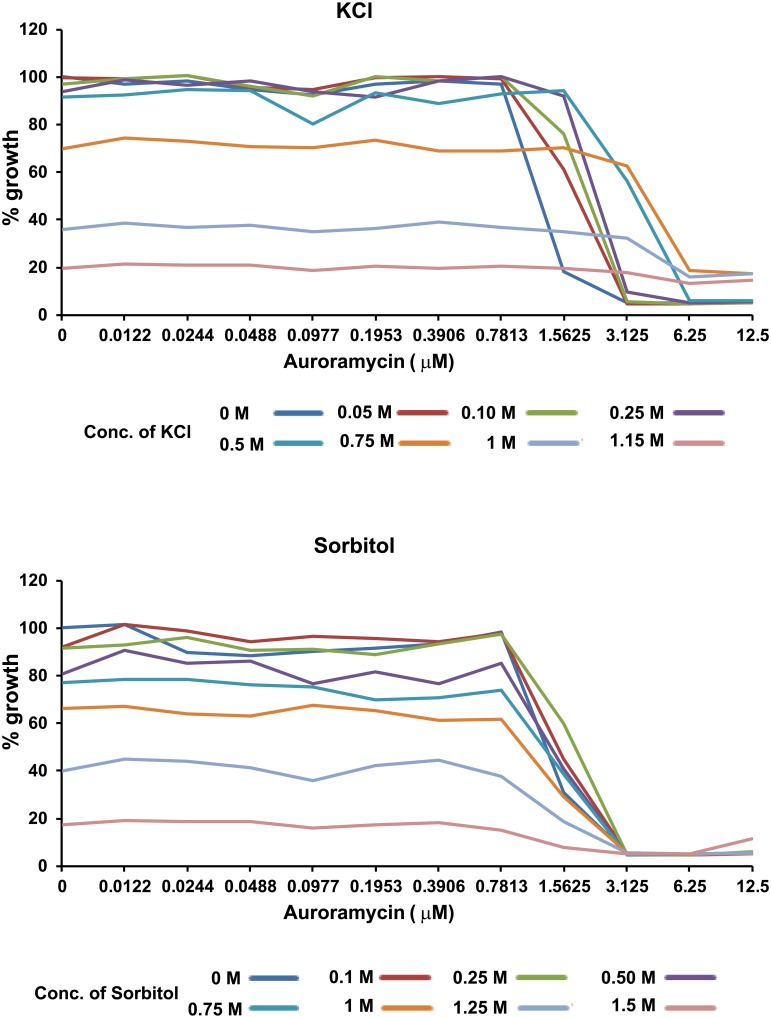
KCl but not sorbitol suppresses the inhibitory effect of auroramycin. Wild type yeast strain (BY4743) was grown in YPD medium containing different concentrations of auroramycin and either KCl or sorbitol at the indicated concentrations in duplicate in a 96-well plate. Average growth of the duplicate cultures at the different concentrations after 16 hours of incubation at 30 °C is plotted. Plots indicating the duplicate OD_600 nm_ values with a picture of the corresponding plate are presented in S5 Fig. Representative data from two independent experiments are shown.

*PMP3* encodes a small conserved membrane protein and its deletion has been shown to increase uptake of cations due to membrane hyperpolarization [28]. Given that *pmp3Δ* cells were resistant to auroramycin (Fig 5; top panel), we hypothesized that auroramycin causes leakage of cations after disrupting membrane integrity. As *pmp3Δ* mutants contain excess of cations [28], they would be more resistant to auroramycin. If this is true, then addition of salt to the medium should suppress the toxic effects of auroramycin. We tested this by treating yeast cells with auroramycin in the presence of either potassium chloride (KCl) or sorbitol at different concentrations. Addition of KCl but not sorbitol rescued the cells from auroramycin-mediated toxicity (Fig 9 and S5 Fig). In contrast, addition of KCl increased the sensitivity to amphotericin B (S6 Fig). These results are consistent with the hypothesis that auroramycin causes leakage of positively charged ions from the cell.

### Auroramycin causes hyperpolarization of yeast cells

**Fig 10:**
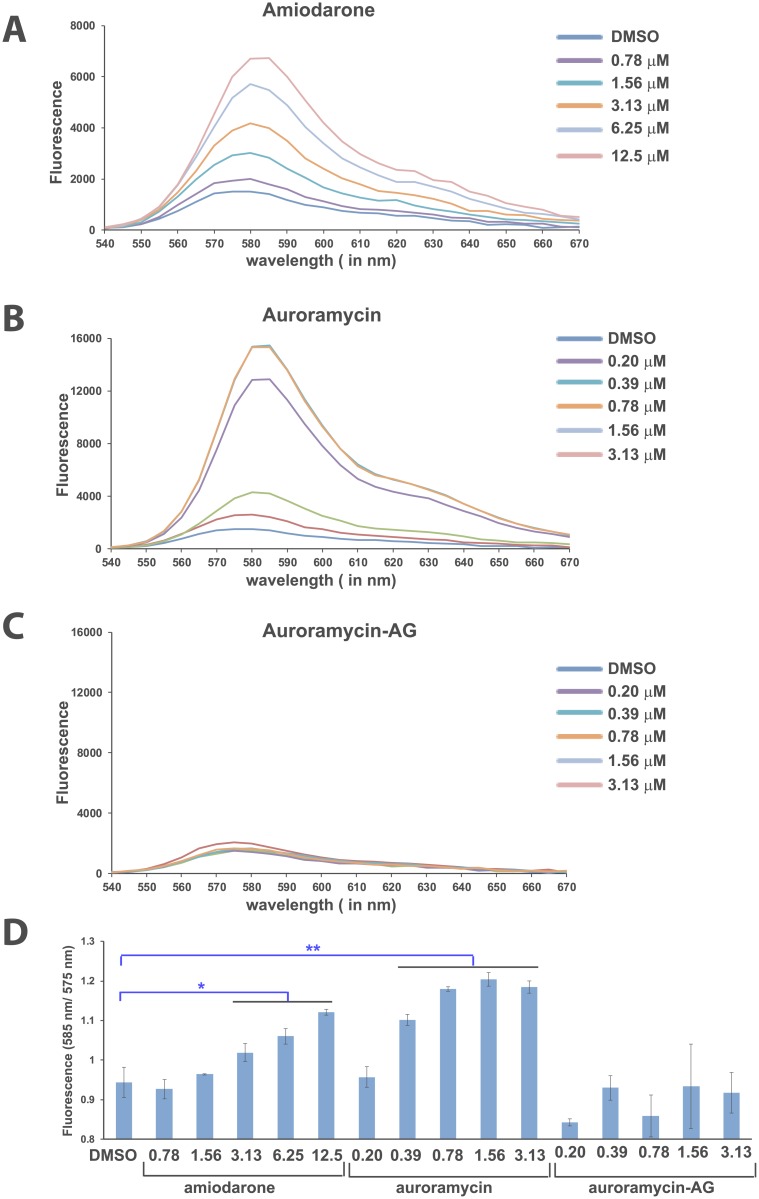
Auroramycin causes hyperpolarization of yeast cells. (A)diS-C_3_(3) fluorescence of wild type AH109 yeast cells suspensions at different concentrations of amiodarone. (B)diS-C_3_(3) fluorescence of wild type AH109 yeast cells suspensions at different concentrations of auroramycin. (C)diS-C_3_(3) fluorescence of wild type AH109 yeast cells suspensions at different concentrations of auroramycin-aglycon. (D)Ratio of fluorescence at 585 nm to 575 nm is plotted for various cultures treated in A-C. Error bars (n = 3 Mean ± S.D) are indicated in the plot. Asterisks indicate statistical significance of fluorescence ratios for amiodarone/auroramycin-treated cells versus DMSO-treated cells, as determined by the Student’s *t* test (**, 2-sided *P* < 0.01 and *, 2-sided P< 0.05). This experiment was performed twice and data from one experiment are shown here.

A possible cause of cation leakage is disruption of membrane potential. To test whether auroramycin affects the membrane potential of yeast cells, we used the potentiometric dye diS-C_3_(3) [29]. This dye is positively charged and its cell entry is favored by negative membrane potential. When diS-C_3_(3) is inside cells, its λ_max_ of emission will shift towards the red end of the spectrum [30]. We used the cationic amphipathic drug amiodarone as a positive control that has been shown to cause membrane hyperpolarization in yeast [31]. We treated yeast cells with either DMSO or auroramycin or auroramycin-aglycon or amiodarone for 20 minutes and then added diS-C_3_ and incubated the cell mixture further for 1 h. We then recorded the diS-C_3_(3) fluorescence emission spectra. Both amiodarone and auroramycin caused a dose-dependent increase in diS-C_3_(3) fluorescence (Fig 10A and 10B). However, the diS-C_3_(3) fluorescence remained largely unaffected in auroramycin-aglycon treated cells (Fig 10C). Red shifts can be computed by increases in λ_max_ emission 585/575 nm ratio. A red shift in fluorescence was observed for amiodarone and auroramycin but not auroramycin-aglycon (Fig 10D).

To confirm that the diS-C_3_(3) dye is internalized by cells following treatment with auroramycin, cells pre-treated with either auroramycin or auroramycin-AG treated with diS-C_3_(3) as described above. We performed flow cytometry and measured the proportion of cells with diS-C_3_(3) fluorescence. Cells treated with auroramycin, displayed increased fluorescence, compared to auroramycin-aglycon treated cells (S7 Fig). Our results are consistent with the hypothesis that auroramycin disrupts membranes and causes cellular leakage of positively charged ions, resulting in hyperpolarized cells that become more permeable to the positively charged diS-C_3_(3).

### Auroramycin works synergistically with cationic drug hygromycin

**Fig 11:**
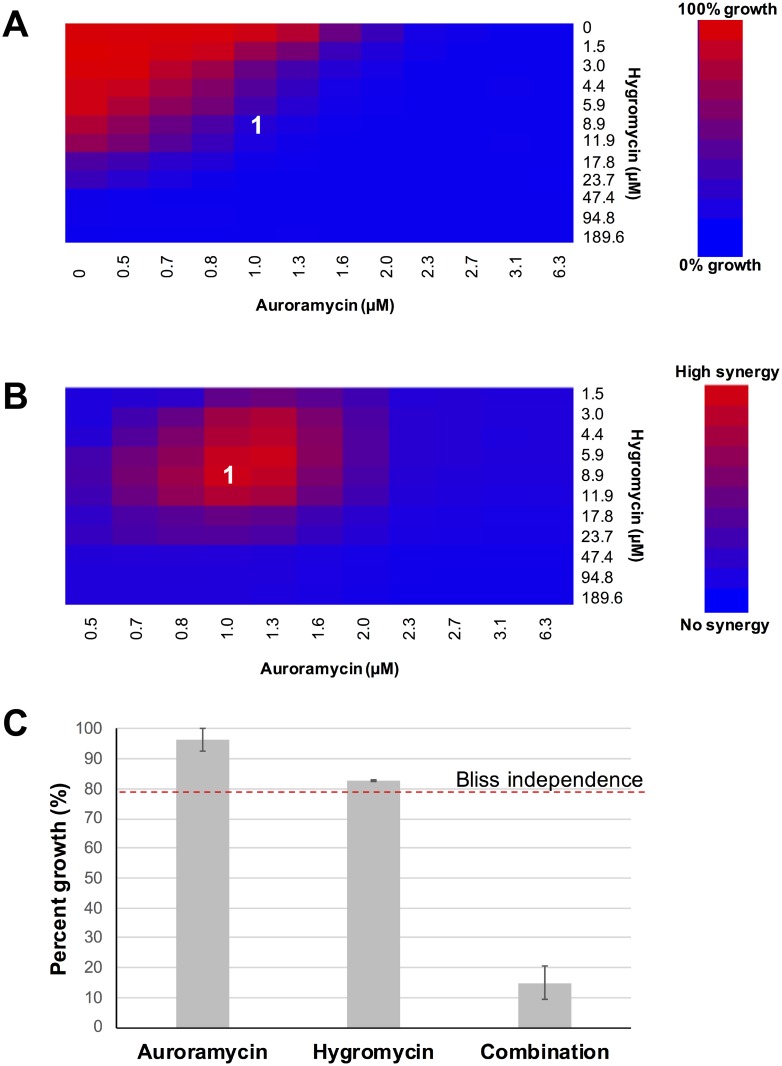
Auroramycin enhances the toxicity of positively charged drug hygromycin. (A)Heatmap for fitness (growth percent) of the dose interaction matrix. (B)Corresponding heatmap for the synergy calculations, based on bliss independence model. Synergy is calculated based on bliss calculations less growth percent. (C)Representative growth rates at 8.9 μM hygromycin and 1.0 μM auroramycin (labelled as (1) in A, B), in comparison to the bliss independence model calculation, P < 0.003. Experiments were performed in triplicates. Error bars (n = 3 Mean ± S.D) are indicated in the plot.

Drugs with complementary actions are often administered as a synergistic combination, which is more efficient compared to the sum of the expected response for individual drugs. Since auroramycin treatment resulted in hyperpolarization of cells, we postulated that this would increase uptake of positively charged molecules such as the cationic drug hygromycin [32–34]. To test for synergistic interactions between auroramycin and hygromycin, a dose interaction matrix assay was conducted and analyzed using a bliss independence model [35]. At lower concentrations, interactions were synergistic based on bliss independent models. Auroramycin enhanced hygromycin’s potency significantly, most likely due to more efficient cationic transportation into the cell (Fig 11).

### Discussion

In this study, we have characterized the antifungal activity of auroramycin, a glycosylated polyene macrolactam. To illuminate its antifungal mode-of-action, a chemogenomic profiling assay in *S*. *cerevisiae* was performed and it identified genes involved in vacuolar transport and membrane organization. We demonstrated that auroramycin disrupts vacuolar organization and membrane permeability via mechanisms that are distinct from amphotericin B, a well-known antifungal. Multiple lines of evidence indicate that their modes of action are different. Firstly, pre-incubation with ergosterol reduces the potency of amphotericin B but not that of auroramycin. Secondly, auroramycin treatment yield defective vacuoles but amphotericin B treatment increased the proportion of cells with single vacuoles compared to controls. Thirdly, salt treatment alleviated the toxicity of auroramycin but exacerbated the growth effect of amphotericin B-treated cells.

Ionic and osmotic stress has been shown to cause vacuolar fragmentation in yeast [36, 37]. Our data indicating that auroramycin causes vacuolar damage and membrane hyperpolarization and that addition of salt rescues auroramycin toxicity strongly suggest that auroramycin causes leakage of cations from yeast cells. It would be interesting to elucidate the molecular mechanism by which auroramycin accomplishes this. One possibility is that auroramycin binds to cellular membranes and forms polar/negatively charged channels that facilitates the efflux of positively charged ions. The presence of polar sugar groups makes it amphipathic which may facilitate its insertion of the molecule in the membrane or/and creation of the polar channel. Alternatively, auroramycin could inhibit proteins that are required directly or indirectly for the uptake of cations from the medium. For example, amiodarone, an ion channel blocker, has been shown to cause hyperpolarization of yeast cells and its toxicity can be suppressed by addition of salt to the medium [31]. Testing if auroramycin causes leakage of cations from artificial membranes such as reconstituted liposomes will help discriminate between these possibilities.

In conclusion, we demonstrated that polyene macrolactam auroramycin’s cellular target and mode-of-action is distinct from those of current polyene macrolactone antifungal agents (23, 24). Auroramycin, in its current form, is less potent as amphotericin B but may be developed as a complement to current antifungal drugs. As auroramycin performs slightly better than amphotericin B in inhibiting *C*. *tropicalis*, an emerging pathogenic strain with growing resistance to fluconazole [38], it could also be a potent second line of therapy. The ability of auroramycin to disrupt membranes and affect membrane potential also presents new opportunities for combinatorial therapies, especially with cationic drugs.

## Methods

### Auroramycin and aglycon purification

Auroramycin and its associated aglycon were fermented and purified from engineered strains of *Streptomyces roseosporus NRRL 15998*, as described previously [3].

### Antifungal assays

Measurements against fungal strains were conducted at Eurofins Panlabs Taiwan (www.eurofins.com/epds), according to methodology described by the Clinical and Laboratory Standards Institute (CLSI) (M38-A, M27-A2).

### Inhibition of growth assays of *S*. *cerevisiae*

The *Saccharomyces cerevisiae* diploid wild-type strain (BY4743) was used for determining the Inhibitory Concentration (IC) of auroramycin. Frozen yeast cells were allowed to recover on YPD agar plates and grown in YPD medium for nine generations (OD_600 nm_ ≤ 2). Cells were diluted to OD_600 nm_ of 0.0625 in YPD medium. 200 μL of the diluted yeast culture was transferred into the 96-well microtiter plate having 2-fold serially diluted concentrations of auroramycin in duplicate. Cells were incubated in a microplate reader for 16–24 hours at 30 °C with shaking. OD_600 nm_ of the cultures was measured using microplate reader Gen 5^TM^ (BIO-TEK Instrument, Vermont, USA). Inhibitory concentration of auroramycin was computed by comparing the growth of treated versus control cells.

### Homozygous profiling (HOP) assay

HOP assay was performed as reported previously [13] with the pooled yeast homozygous Knock out collection (Invitrogen). Auroramycin was used at 1.56 μM which caused a 50% reduction in growth in YPD-HEPES medium after 10 hours at 30 °C. Preparation of genomic DNA from yeast cells, amplification of barcode sequences by PCR and Next Generation Sequencing (NGS) of the PCR products were all performed as previously described [14].

### Bioinformatic methods

Analysis of NGS data and identification of differentially-sensitive strains was performed as recently described [15]. The sequenced reads were demultiplexed and the resulting raw fastq reads were processed as follows. Firstly, reads without the adaptor sequence (GTCCACGAGGTCTCT) that flank the uptag barcode were filtered out. After extracting the uptag barcodes, the identical sequences were enumerated and collapsed. The TAG counts were then aligned to a library of TAG sequences to connect each read count to the deletion strain using the Novoalign software (http://novocraft.com V2.05.33) with default settings (-n 22 to truncate the reads to the length of the tag barcode). This setting permitted up to a single base pair mismatch. The resulting SAM (Sequence Alignment Map) files were parsed and the total counts of each TAG that correspond to each homozygous deletion strain were determined. The EdgeR analysis package [39] was utilised to analyse the counts using the generalized linear model (glm) mode to contrast the treated samples to the control (DMSO-treated) sample and differentially-sensitive strains were identified. The generalized linear models (glm) were used to compute the trended and tagwise dispersion using the *estimatedGLMTrendedDisp* and *estimateGLMTagwiseDisp* functionality. A likelihood ratio test was then used (glmFIT() and glmLRT()) to test for differential sensitivity. This generated a log fold-change ratio of tags between treated and control samples. A p-value threshold of p<0.05 was used to identify mutants with significant growth defect and were used for further analysis.

Gene Ontology analysis was performed using the online tools DAVID [16, 17] and REVIGO [18].

### FM 4–64 labelling of vacuoles

Yeast cells were labelled with FM4-64 (Invitrogen) as previously reported [40]. Overnight culture of BY4743 strain was diluted into fresh nutrient medium at OD_600 nm_ = 0.2 and grown for approximately 4 hours until OD_600 nm_ = 0.8. Cells were labelled with 15 μM FM4-64 in YPD for 1 hour, at 30 °C with shaking. After that, the cells were washed and treated with either DMSO or various concentrations of either auroramycin or auroramycin-aglycon or amphotericin B for 30 minutes, at 30 °C with shaking. Following that, the cells were spun down at 3000 *g* for 5 minutes and washed once with 1 mL of Synthetic Defined (SD) media. After that, the cells were resuspended in 30 μL of SD media. The images were acquired using an inverted fluorescence microscope ZEISS LSM 5 LIVE (Carl Zeiss, Oberkochen, Germany).

### CFDA leakage assay

Non-polar CFDA-SE spontaneously enters into the cell and is converted to the anionic pH-sensitive 5-(and-6)-carboxyfluorescein succinimidyl ester (CF-SE) by intracellular esterases. After internalization, amine reactive coupling of succinimidyl groups of CF-SE to aliphatic amines of intracellular proteins and forms membrane-impermeable pH-sensitive probe conjugates [26]. Overnight culture of yeast cells was diluted into fresh medium at a starting of OD_600 nm_ = 0.2 and incubated for few hours until OD_600 nm_ reached 0.8. Cells were collected by centrifugation at 3000 *g* for 3 min. After that, the cells were washed and resuspended in an equal volume of citric/phosphate buffer (100 mM citric acid, 50 mM NaH_2_PO_4_, and 50 mM KOH; pH 4) containing 100 μM of CFDA-SE (Cayman Chemical). Cells were then incubated overnight at 37 °C with shaking. Next day, cells were collected by centrifugation at 3000 *g* for 3 min, washed and resuspended in synthetic defined (SD) medium with citric/phosphate buffer (pH 4) at OD_600 nm_ = 0.4. Cells were incubated at 30 °C, 1 hour with shaking. After that cells were either treated with DMSO or various concentrations of auroramycin or amphotericin B. and further incubated at 30 °C with shaking. Samples of the culture were taken after 30’ and 120’ and centrifuged to pellet the cells. Fluorescence reading in the supernatant was measured at 525 nm (excitation at 495 nm) using microplate reader Gen 5^TM^ (BIO-TEK Instrument, Vermont, USA).

### Ergosterol competition assay

Different concentrations of ergosterol (2.5 mM stock in DMSO) or DMSO was premixed in YPD-HEPES media that contained different concentrations of amphotericin B, auroramycin or DMSO in a polystyrene 96-well plate [35]. Subsequently, BY4743 cells were added at OD_600 nm_ = 0.0625 into the wells and the plates were incubated overnight at 30 °C. OD_600 nm_ was measured after 16 h using a microplate reader Gen 5^TM^ (BIO-TEK Instrument, Vermont, USA).

### Hygromycin and auroramycin dose interaction matrix assay

BY4743 cells from an overnight culture were diluted to OD_600 nm_ of 0.0625 in YPD-HEPES medium. 200 μL of the diluted yeast culture was transferred into the 96-well microtiter plate having various concentrations of auroramycin and hygromycin. Cells were incubated in a microplate reader for 16–24 hours at 30 °C with shaking. Growth of the cultures was recorded by measuring the OD_600 nm_ using the microplate reader Gen 5^TM^ (BIO-TEK Instrument, Vermont, USA). The bliss model assumes that the responses are independent events, such as distinct mode-of-actions, and uses a probabilistic calculation (E_A_+E_B_(1-E_A_)) to an expected combined effect of drug A(E_A_) and drug B (E_B_). Synergy is given by the percent excess of the bliss calculation. Heatmaps were generated using Shinyheatmap [41].

### Membrane potential diS-C_3_(3) assay

Membrane potential of yeast cells using diS-C_3_(3) was measured as previously described with minor modifications [29]. Wild type AH109 yeast cells from an overnight culture were diluted into YPD medium at an OD_600 nm_ = 0.2 and grown for few hours until the OD reached 1.0. Cells were harvested, washed once with 10 mM citric/phosphate buffer (10 mM citric acid, 5 mM NaH_2_PO_4_, and 5 mM KOH, pH4). Then, the cells were resuspended in 10 mM citric/phosphate buffer at OD_600 nm_ = 1.0 and treated with DMSO, different concentrations of amiodarone or auroramycin or aglycon for 20 min, at 30 °C with shaking. diS-C_3_(3) (3,30-dipropylthiacarbocyanine iodide) fluorescence probe (1 mM stock solution in ethanol) was added to 2 mL of yeast cell suspension to a final concentration of 0.5 μM. Cells were incubated with the dye for 1 hour at room temperature with shaking. For each sample, the diS-C_3_(3) fluorescence emission spectra of the suspensions were recorded and λ_max_ was assessed using microplate reader Gen 5^TM^ (BIO-TEK Instrument, Vermont, USA). For flow cytometric analysis, cells were washed with phosphate-buffered saline (PBS) and resuspended in PBS. Flow cytometry was performed on a Becton Dickinson (BD) FACSAria Fusion (Becton Dickinson, Oxford UK) using the PE-A channel.

## Supporting information

S1 Fig Auroramycin displays cytotoxic effect on yeast cells. *Saccharomyces cerevisiae* strain BY4743 cultures were treated either cycloheximide (1 μM) or amphotericin B (1.56 μM) or auroramycin (6.25 μM) or solvent (water/ DMSO) for 24 hours. Cells were then washed with YPD and plated at different dilutions on YPD agar. Photographs of the YPD agar plate were taken after 2 days at 30 °C. Cells treated with amphotericin B and auroramycin fail to recover on YPD agar plates in contrast to cells treated with the cytostatic inhibitor cycloheximide.
(PDF)

S2 Fig Calculation of IC_50_ value for auroramycin against wild type yeast cells. Experimental data presented in Fig 2 was used to calculate the IC_50_ value of auroramycin. Percentage growth was plotted against log (concentration of auroramycin in μM). The IC_50_ value was determined by a variable slope dose-response curve using the GraphPad prism software.
(PDF)

S3 Fig Calculation of IC_50_ values for auroramycin and amphotericin B against wild type yeast cells. Experimental data presented in Fig 2 was used to calculate the IC_50_ value of auroramycin. Percentage growth was plotted against log (concentration of auroramycin or amphotericin B in μM). The IC_50_ value was determined by a variable slope dose-response curve using the GraphPad Prism software.
(PDF)

S4 Fig Auroramycin’s inhibitory effect is not *via* interaction with ergosterol. Plots in Fig 6 containing vertical bars that represent the duplicate OD_600 nm_ values are presented in A (auroramycin) and B (amphotericin B) along with an image of the corresponding 96-well plate on the right.
(PDF)

S5 Fig KCl but not sorbitol suppresses the inhibitory effect of auroramycin. Plots in Fig 9 containing vertical bars that represent the duplicate OD_600 nm_ values are presented in A (KCl) and B (Sorbitol) along with an image of the corresponding 96-well plate on the right.
(PDF)

S6 Fig KCl enhances the inhibitory effect of amphotericin B. Wild type yeast strain (BY4743) was grown in YPD medium containing different concentrations of amphotericin B and KCl at the indicated concentrations in duplicate in a 96-well plate. Average growth of the duplicate cultures at the different concentrations after 16 hours of incubation at 30 °C is plotted.
(PDF)

S7 Fig Auroramycin causes hyperpolarization of yeast cells. Flow cytometric analyses of various combinations of cells with dye, DMSO, auroramycin, and aglycon. This experiment was performed twice and data from one experiment are shown here.
(PDF)

S1 Table HOP data of auroramycin and GO analysis of genes conferring resistance to auroramycin. (XLSX)
