# Correction: Chemogenomic profiling in yeast reveals antifungal mode-of-action of polyene macrolactam auroramycin

**DOI:** 10.1371/journal.pone.0221074

**Published:** 2019-08-08

**Authors:** Jin Huei Wong, Mohammad Alfatah, Kiat Whye Kong, Shawn Hoon, Wan Lin Yeo, Kuan Chieh Ching, Corinna Jie Hui Goh, Mingzi M. Zhang, Yee Hwee Lim, Fong Tian Wong, Prakash Arumugam

Fig 10 is incorrect. Please see the correct Fig 10 here.

**Fig 10:**
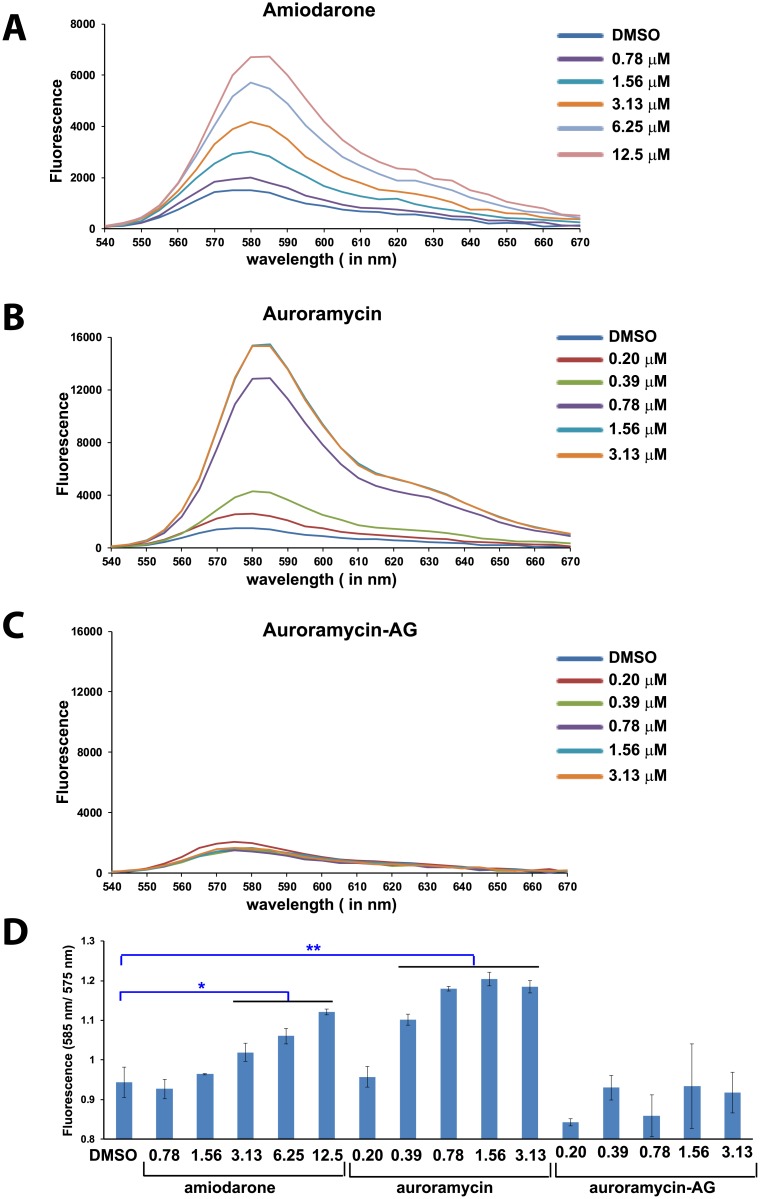
Auroramycin causes hyperpolarization of yeast cells. (A)diS-C_3_(3) fluorescence of wild type AH109 yeast cells suspensions at different concentrations of amiodarone. (B)diS-C_3_(3) fluorescence of wild type AH109 yeast cells suspensions at different concentrations of auroramycin. (C)diS-C_3_(3) fluorescence of wild type AH109 yeast cells suspensions at different concentrations of auroramycin-aglycon. (D)Ratio of fluorescence at 585 nm to 575 nm is plotted for various cultures treated in A-C. Error bars (n = 3 Mean ± S.D) are indicated in the plot. Asterisks indicate statistical significance of fluorescence ratios for amiodarone/auroramycin-treated cells versus DMSO-treated cells, as determined by the Student’s *t* test (**, 2-sided *P* < 0.01 and *, 2-sided P< 0.05). This experiment was performed twice and data from one experiment are shown here.
