# Supplementary figures and images for: Chemogenomic profiling in yeast reveals antifungal mode-of-action of polyene macrolactam auroramycin
Source: PLoS One. 2019 Jun 10;14(6):e0218189. doi: 10.1371/journal.pone.0218189 (PMC6557514; doi:10.1371/journal.pone.0218189)

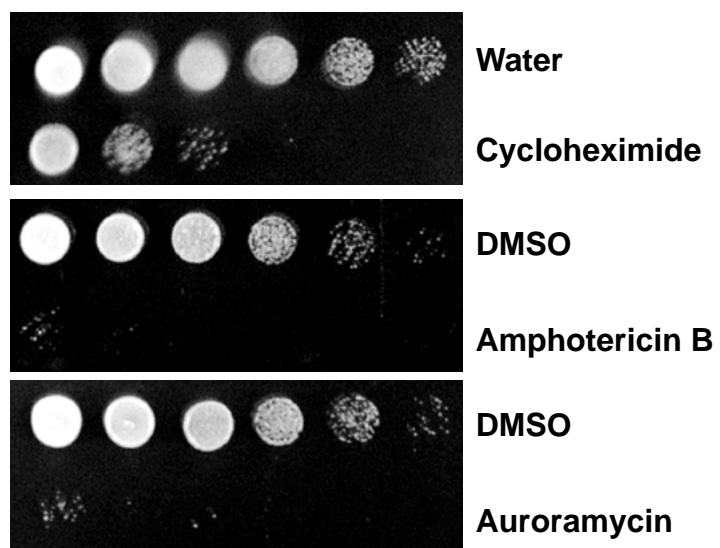

**Fig S1**

Supplement: S1 Fig — Saccharomyces cerevisiae strain BY4743 cultures were treated either cycloheximide (1 μM) or amphotericin B (1.56 μM) or auroramycin (6.25 μM) or solvent (water/ DMSO) for 24 hours. Cells were then washed with YPD and plated at different dilutions on YPD agar. Photographs of the YPD agar plate were taken after 2 days at 30 °C. Cells treated with amphotericin B and auroramycin fail to recover on YPD agar plates in contrast to cells treated with the cytostatic inhibitor cycloheximide. (PDF) [file pone.0218189.s001.pdf]

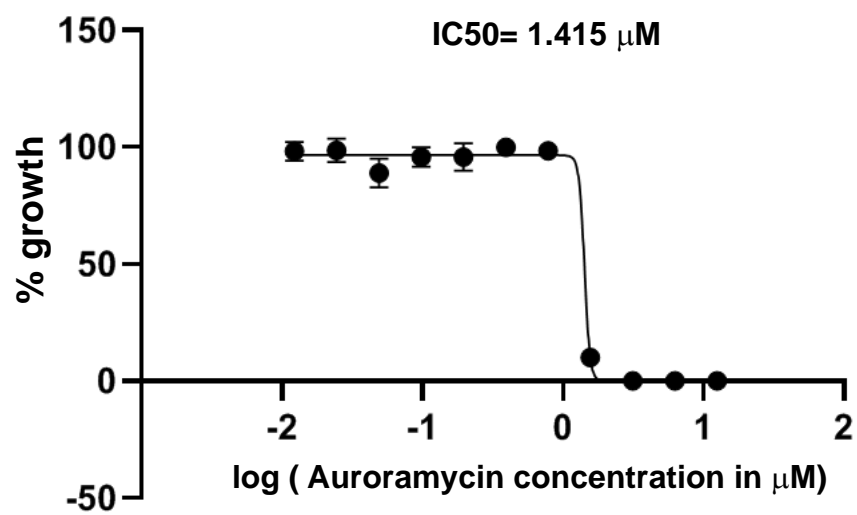

**Fig S2**

Supplement: S2 Fig — Experimental data presented in Fig 2 was used to calculate the IC50 value of auroramycin. Percentage growth was plotted against log (concentration of auroramycin in μM). The IC50 value was determined by a variable slope dose-response curve using the GraphPad prism software. (PDF) [file pone.0218189.s002.pdf]

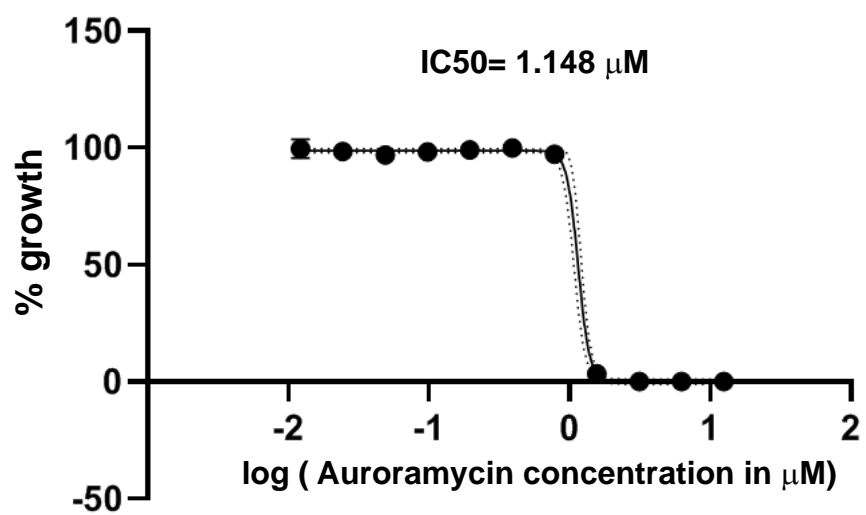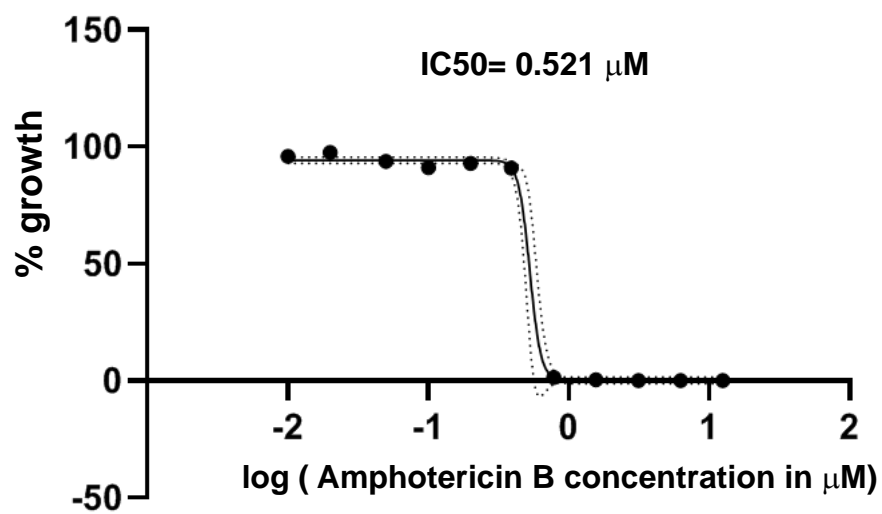

**Fig S3**

Supplement: S3 Fig — Experimental data presented in Fig 2 was used to calculate the IC50 value of auroramycin. Percentage growth was plotted against log (concentration of auroramycin or amphotericin B in μM). The IC50 value was determined by a variable slope dose-response curve using the GraphPad Prism software. (PDF) [file pone.0218189.s003.pdf]

**A**

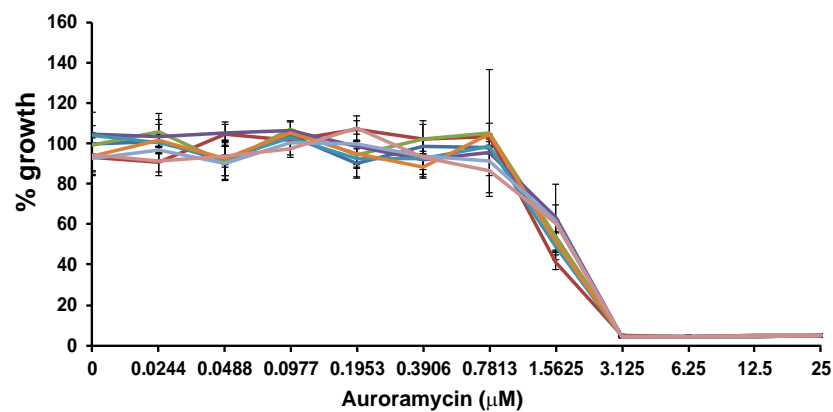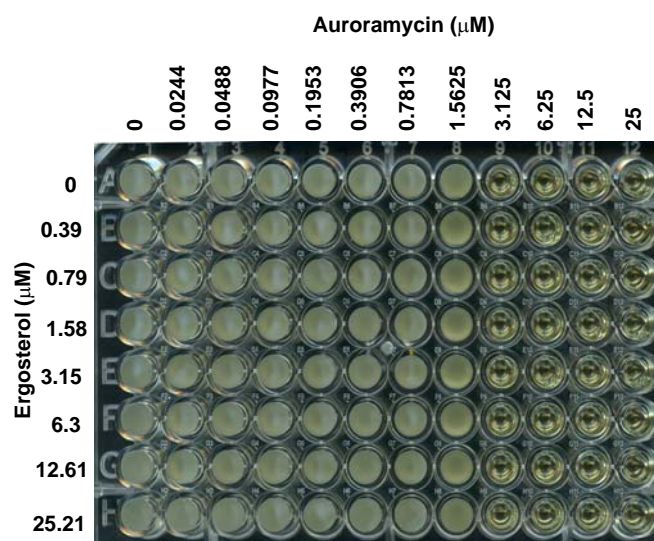

**B**

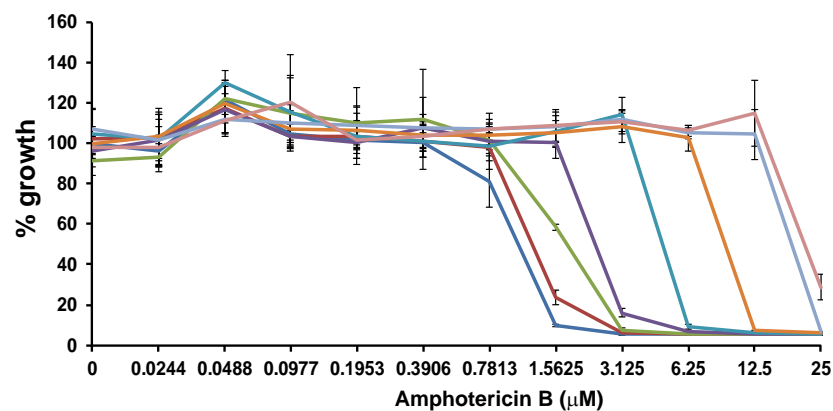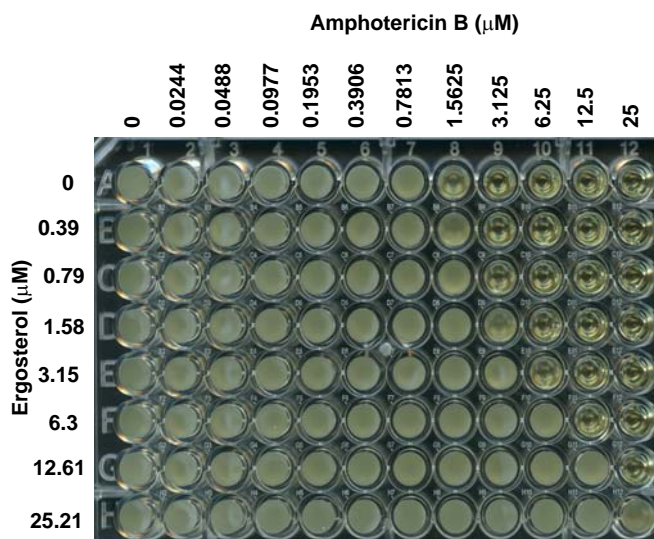

**Ergosterol ( $\mu\text{M}$ )**

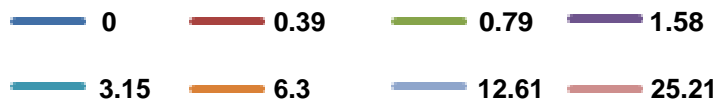

**Fig S4**

Supplement: S4 Fig — Plots in Fig 6 containing vertical bars that represent the duplicate OD600 nm values are presented in A (auroramycin) and B (amphotericin B) along with an image of the corresponding 96-well plate on the right. (PDF) [file pone.0218189.s004.pdf]

**A**

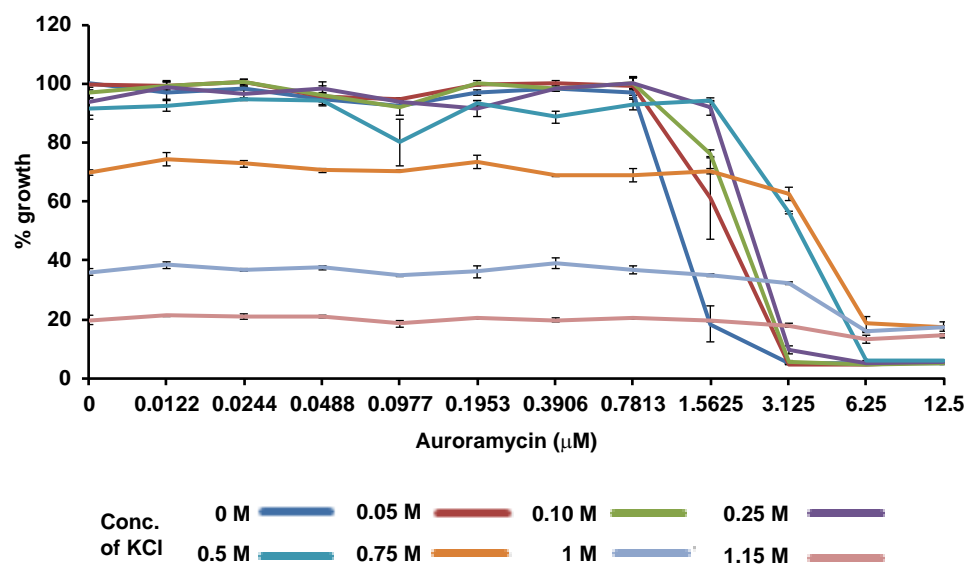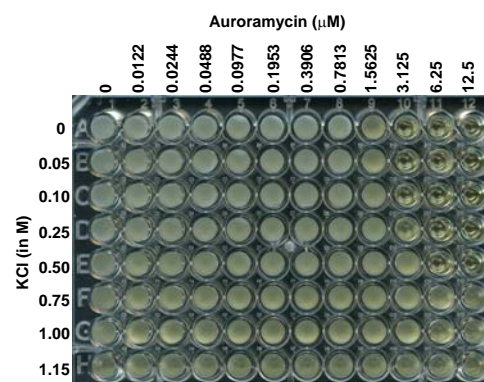

**B**

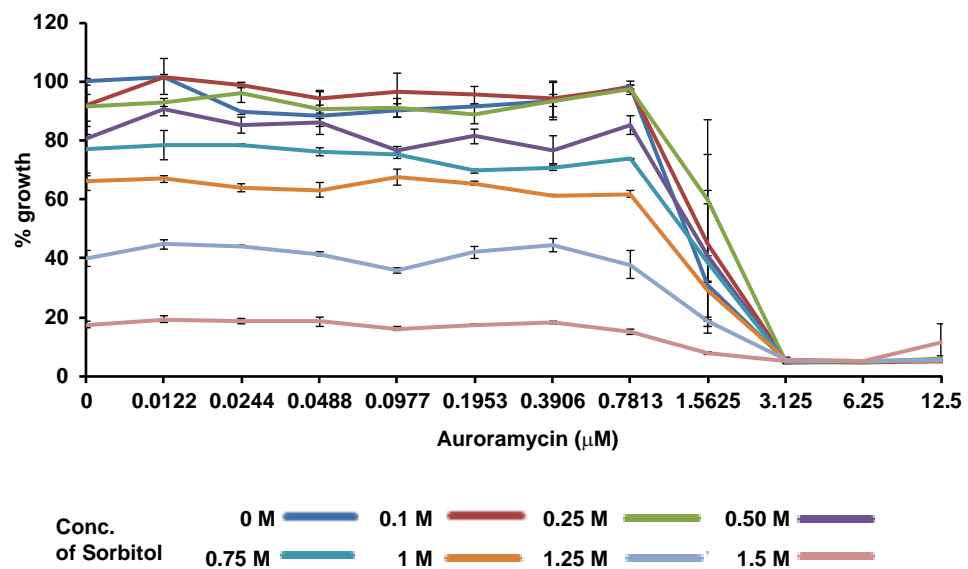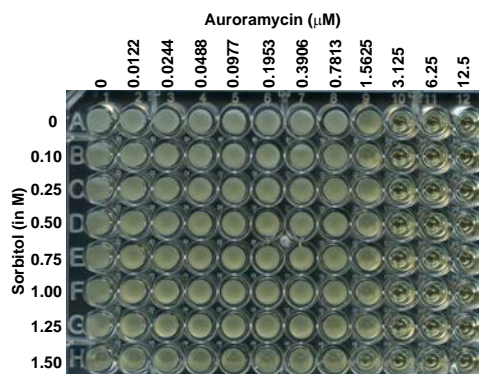

**Fig S5**

Supplement: S5 Fig — Plots in Fig 9 containing vertical bars that represent the duplicate OD600 nm values are presented in A (KCl) and B (Sorbitol) along with an image of the corresponding 96-well plate on the right. (PDF) [file pone.0218189.s005.pdf]

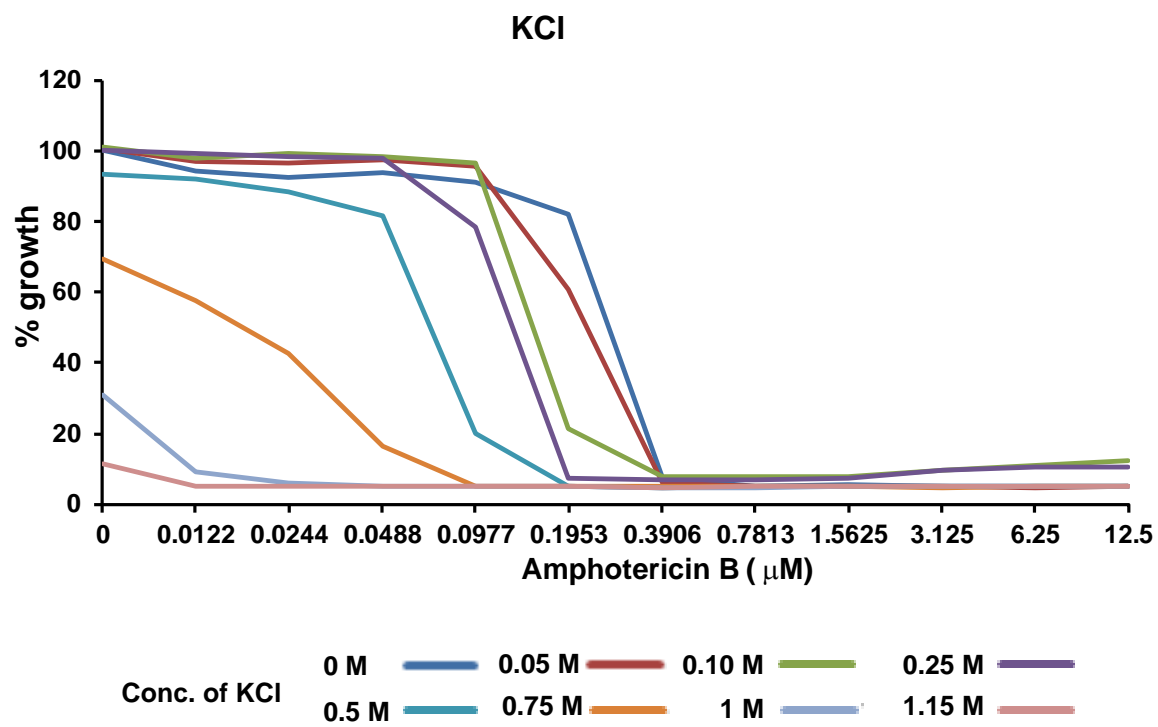

**Fig S6**

Supplement: S6 Fig — Wild type yeast strain (BY4743) was grown in YPD medium containing different concentrations of amphotericin B and KCl at the indicated concentrations in duplicate in a 96-well plate. Average growth of the duplicate cultures at the different concentrations after 16 hours of incubation at 30 °C is plotted. (PDF) [file pone.0218189.s006.pdf]

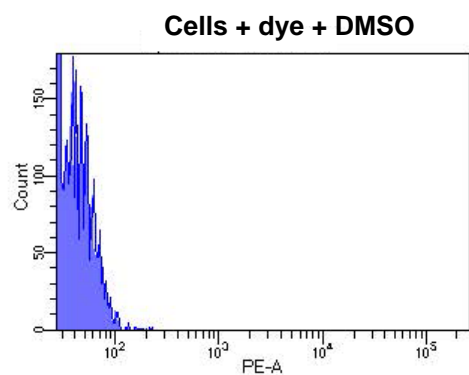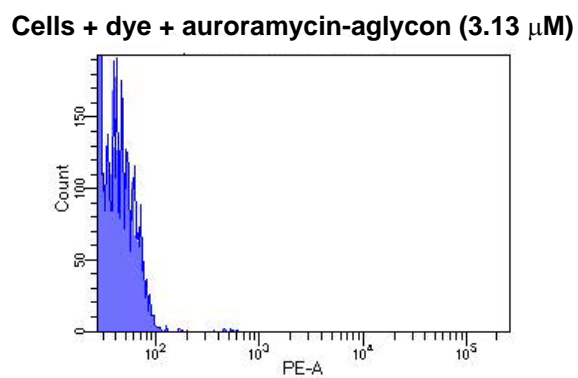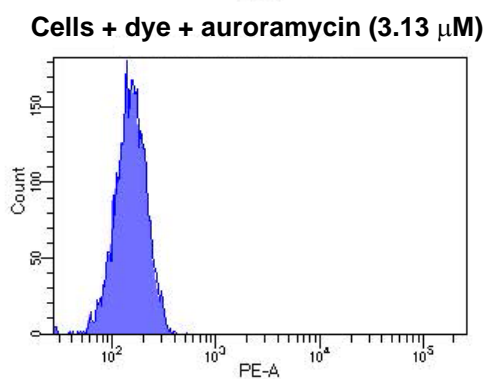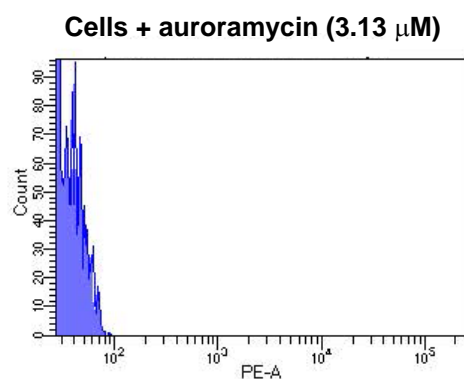

**Fig S7**

Supplement: S7 Fig — Flow cytometric analyses of various combinations of cells with dye, DMSO, auroramycin, and aglycon. This experiment was performed twice and data from one experiment are shown here. (PDF) [file pone.0218189.s007.pdf]
